# Benchmark of Approximate Quantum Chemical and Machine Learning Potentials for Biochemical Proton Transfer Reactions

**DOI:** 10.1021/acs.jctc.5c00690

**Published:** 2025-06-30

**Authors:** Guilherme M. Arantes, Jan Řezáč

**Affiliations:** † Instituto de Estudos Avançados, Universidade de São Paulo, Rua da Praça do Relógio 109, São Paulo, SP 05508-050, Brazil; ‡ Instituto de Química, Universidade de São Paulo, Av. Prof. Lineu Prestes 748, São Paulo, SP 05508-900, Brazil; § Institute of Organic Chemistry and Biochemistry, Czech Academy of Sciences, Prague 160 00, Czech Republic

## Abstract

Proton transfer reactions are among the most common chemical transformations and are central to enzymatic catalysis and bioenergetic processes. Their mechanisms are often investigated using DFT or approximate quantum chemical methods, whose accuracy directly impacts the reliability of the simulations. Here, a comprehensive set of semiempirical molecular orbital and tight-binding DFT approaches, along with recently developed machine learning (ML) potentials, are benchmarked against high-level MP2 reference data for a curated set of proton transfer reactions representative of biochemical systems. Relative energies, geometries, and dipole moments are evaluated for isolated reactions. Microsolvated reactions are also simulated using a hybrid QM/MM partition. Traditional DFT methods offer high accuracy in general but show markedly larger deviations for proton transfers involving nitrogen-containing groups. Among approximate models, RM1, PM6, PM7, DFTB2-NH, DFTB3, and GFN2-xTB show reasonable accuracy across properties, though their performance varies by chemical group. The ML-corrected (Δ-learning) model PM6-ML improves accuracy for all properties and chemical groups and transfers well to QM/MM simulations. Conversely, standalone ML potentials perform poorly for most reactions. These results provide a basis for evaluating approximate methods and selecting potentials for proton transfer simulations in complex environments.

## Graphical abstract

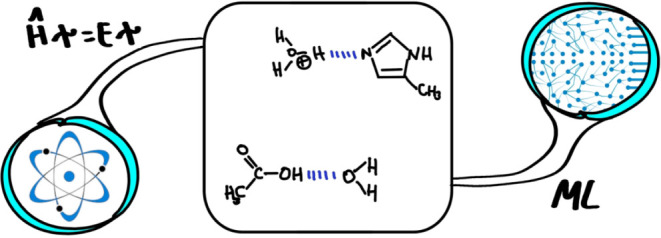

## Introduction

Proton transfer (PT) reactions are among the most fundamental chemical processes. They play a central role in acid–base chemistry, catalysis, and hydrogen-bond dynamics. In biochemistry, PT reactions are ubiquitous and underpin enzymatic mechanisms, p*K*_*a*_ regulation, and energy transduction pathways, such as those catalyzed by respiratory complexes in the mitochondrial electron transport chain.^1^

Computational modeling of PT reactions in condensed-phase or enzymatic systems is typically performed using hybrid quantum mechanics/molecular mechanics (QM/MM) potentials, which allow for a quantum treatment of the reactive centers embedded in a classical electrostatic environment.^2–4^ However, applications of accurate quantum chemical (QC) methods such as DFT or post-Hartree–Fock approaches with hybrid potentials remain computationally demanding. This limits the size of the QM region, as well as the length and number of simulations—an issue that is particularly problematic because proton wires and hydrogen-bond networks reorganize dynamically and respond to nonlocal structural fluctuations.^5,6^

To address these challenges, approximate quantum methods such as semiempirical molecular orbital models,^7^ empirical valence-bond,^8^ tight-binding DFT approximations,^9,10^ and more recently, machine learning (ML)-based potentials^11–13^ can in principle be used to enable longer sampling times while maintaining a quantum mechanically compatible description of the reactive center. These methods offer a compromise between accuracy and computational efficiency, and are especially useful when simulating rare proton transfer events over long time scales or multiple putative mechanisms.

Calibration of approximate or lower-level quantum methods toward specific chemical systems is an effective strategy to improve simulation accuracy. Targeted parametrizations may allow the refinement of models to reproduce references for particular classes of reactions. One of us applied this approach to develop specific semiempirical parametrizations for PT coupled to phosphate reactions,^14^ halogenations^15^ and hydrolysis of iron–sulfur clusters,^16,17^ where standard methods typically fail. Similar efforts have been made for liquid water to improve the accuracy of hydrogen bonding and structural properties.^18,19^ However, these water-specific calibrations were limited to neutral molecules and were not extended to other protonable groups. This reparametrization approach can also be extended to machine learning, where a general-purpose ML potential may be fine-tuned to improve performance for specific PT reactions or chemical environments.^13,20^

Previous benchmark studies have evaluated quantum methods (mainly DFT) for modeling proton transfer reactions.^21–24^ However, they were limited to small reaction sets or specific systems. In the past decade, many new approximate QC methods and parametrizations have been introduced, often claiming broad applicability and improved accuracy.^25–28^ Yet, their performance for modeling PT reactions, especially in the context of biochemical systems and hybrid QM/MM potentials, remains insufficiently tested.

In this work, we present a comprehensive benchmark of approximate quantum methods and ML potentials for modeling proton transfer reactions across eight biologically relevant chemical groups, representing all amino acids with protonable side chains, bioenergetically important quinones, and water molecules. We evaluate the accuracy of these methods in reproducing isolated relative energies, geometries and dipole moments, as well as their performance when used in QM/MM potentials for microsolvated reactions surrounded by a few water molecules. Our results can guide method selection for realistic and long time scale simulations of proton transfer in complex environments.

## Computational Methods

### Benchmark Calculations and Tested Methods

*Ab initio* calculations at the MP2 level^29^ with the def2-TZVP basis set^30^ were used as references for all properties: relative energies (Tables 2, 3 and 5), dipole moments (Table S1) and geometries (Table 4). This level of theory is sufficiently accurate for benchmarking the approximate methods assessed here. For instance, the difference in mean unsigned error computed with MP2 and with the more accurate but computationally demanding CCSD­(T) method, both using the same triple-ζ basis set, is less than 4 kJ/mol for a set of 17 isogyric reaction energies^29^ and below 1 kJ/mol for a set of 8 proton transfer relative energies.^23^

**Table 1:** Proton Transfer Reactions Studied Here with *N* Steps or Geometries along the Transfer Pathway, with Fixed Distance between Proton Donor (D) and Acceptor (A) Atoms^a^

Name	Reaction	*N*	Distance (Å)
Amine1	CH_3_NH_3_^+^ + H_2_O	9	2.5
Amine2	CH_3_NH_3_^+^ + OH^–^	9	3.1
Carboxylate1	CH_3_COOH + H_2_O	7	2.7
Carboxylate2	CH_3_COOH + OH^–^	10	3.0
Carboxylate3	CH_3_COOH + 2 H_2_O	14	(2.6, 2.7)
Carboxylate4	CH_3_COOH + H_2_O + H_3_O^+^	5	(2.6, 2.4)
Guanidine1	CH_3_NH(C^+^)(NH_2_)_2_ + H_2_O	9	2.8
Guanidine2	CH_3_NH(C^+^)(NH_2_)_2_ + OH^–^	10	3.1
Guanidine3	CH_3_NH(C^+^)(NH_2_)_2_ + 2H_2_O	8	2.8
Imidazole1	CH_3_Imz + H_3_O^+^	8	3.0
Imidazole2	CH_3_Imz + H_2_O	11	3.2
Imidazole3	CH_3_Imz + OH^–^	9	3.0
Imidazole4	CH_3_Imz + H_3_O^+^	8	3.0
Phenol1	C_6_H_5_OH + H_2_O	9	2.6
Phenol2	C_6_H_5_OH + OH^–^	8	2.5
Phenol3	C_6_H_5_OH + H_3_O^+^	6	2.5
Quinone1	Q + H_2_O	6	2.8
Quinone2	QH_2_ + OH^–^	8	2.5
Quinone3	QH_2_ + H_2_O	7	2.5
Quinone4	Q + H_3_O^+^	9	2.5
Quinone5	QH^•^ + H_2_O	9	2.5
Thiol1	CH_3_SH + H_2_O	9	2.8
Thiol2	CH_3_SH + H_3_O^+^	9	2.8
Eigen1	H_3_O^+^ + 3 H_2_O	7	2.7
Eigen2	H_3_O^+^ + 3 H_2_O	8	2.9
Zundel1	H_3_O^+^ + H_2_O	9	2.5
Zundel2	H_3_O^+^ + H_2_O	11	2.7
Zundel3	H_3_O^+^ + H_2_O	14	2.9
Zundel4	H_3_O^+^ + H_2_O	13	3.2
WaterWire	H_3_O^+^ + 2 H_2_O + OH^–^	8	2.7
Total		267	

a Figure S3 shows the orientation and D–A atoms. CH_3_Imz is 4-methyl-imidazole, Q is 2,3-dimethoxy-5-methyl-1,4-benzoquinone, and QH_2_ is the equivalent benzene-1,4-diol.

**Table 2:** Mean Unsigned Error (MUE, in kJ/mol) of Relative Energies Calculated with Quantum Chemical Methods in Comparison to the MP2 Reference for the Complete Set of Isolated Proton Transfer Reactions (Table 1)­^a^

	Chemical group								
Method	–NH_3_	COOH	^+^CNH_2_	=NH	PhOH	Q	–SH	H_2_O	Avg
MNDO	81.5	75.1	69.0	47.7	61.1	67.2	21.7	44.7	57.5
pddgMNDO	69.4	61.1	51.2	40.4	50.7	46.9	NA^b^	33.2	46.6
AM1	42.9	38.7	26.2	28.3	44.3	52.3	29.5	23.2	35.0
PM3	26.6	34.2	12.8	11.5	37.7	47.4	22.4	21.3	27.6
pddgPM3	26.7	17.7	17.3	17.7	24.9	24.1	29.3	17.1	21.0
RM1	15.0	11.0	9.91	11.5	18.2	18.8	16.6	15.4	**14.8**
PM6	15.7	22.7	25.4	16.1	15.2	23.1	24.2	18.2	20.3
PM6-ORG	23.1	19.4	23.1	29.3	13.4	17.7	31.8	12.4	19.6
PM6-D3H4	12.3	14.3	21.5	10.5	8.44	15.8	24.2	20.5	16.2
PM7	13.0	10.3	14.1	7.03	10.2	14.1	27.6	15.7	**13.4**
noSCC	119	37.3	48.2	49.2	52.3	43.6	26.7	59.0	54.2
DFTB2	44.6	9.30	21.7	13.2	16.9	20.8	17.9	12.1	17.5
DFTB2-NH	26.7	9.30	24.1	14.3	16.9	20.8	17.9	12.1	16.7
DFTB3	14.4	5.74	23.1	30.1	20.8	20.7	4.65	5.70	15.2
DFTB3-NH	15.3	5.74	38.5	51.3	20.8	20.7	4.65	5.70	19.7
GFN1-xTB	16.7	10.7	17.5	17.2	15.4	19.6	25.4	9.02	15.2
GFN2-xTB	22.2	10.0	13.0	11.7	9.70	20.1	5.60	12.2	**13.5**
UAIQM^c^	27.6	16.4	21.8	13.7	22.3	23.1	19.4	6.88	17.0
PM6-ML	7.26	15.1	9.38	10.3	5.92	14.7	14.8	8.13	**10.8**
BLYP	11.3	13.8	14.3	22.7	8.39	18.1	7.41	16.5	15.7
PBE	11.9	14.7	15.1	23.3	10.4	18.3	10.1	15.5	16.1
M06L	6.99	3.94	3.82	10.1	9.19	15.7	3.33	8.06	8.35
B3LYP-3G	26.3	23.7	19.7	32.7	30.3	25.4	37.5	25.1	26.8
B3LYP	7.29	5.41	4.73	9.54	7.15	11.4	4.07	8.94	7.93
ωB97X	8.04	4.44	3.99	4.04	8.11	8.22	1.22	7.87	**6.16**

a Columns give the average MUE among amine (−NH_3_), carboxylate (COOH), guanidine (^+^CNH_2_), imidazole (=NH), phenol (PhOH), quinone (Q), thiol (−SH) and collectively Zundel, Eigen and WaterWire (H_2_O) water cluster reactions. Avg is the average MUE for all 30 reactions and bold font is used to indicate the best performance within each group of methods.

b NA = not applicable.

c UAIQM/GFN2-xTB.

**Table 3:** MUE (kJ/mol) of Relative Energies Calculated with Methods Composed by ML Potentials for a Subset of 8 Neutral Reactions: Amine2 (−NH_3_), Carboxylate1 (COOH), Guanidine2 (^+^CNH_2_), Imidazole2 (=NH), Phenol1 (PhOH), Quinone1 (Q), Thiol1 (−SH) and WaterWire (H_2_O, Table 1)­^a^

	Chemical group								
Method	–NH_3_	COOH	^+^CNH_2_	=NH	PhOH	Q	–SH	H_2_O	Avg
ANI-1x-D4	92.2	12.5	43.5	26.7	23.7	34.2	NA	190	60.4
ANI-2x-D4	40.3	8.73	20.2	25.7	22.6	24.7	5.54	131	34.9
ANI-1ccx	89.2	10.0	39.6	25.2	21.0	37.4	NA	221	63.3
ANI-1xnr	50.3	2.49	17.8	66.0	46.5	20.6	NA	141	49.3
MACE-OFF23-L	44.6	22.6	23.0	31.0	17.5	10.9	3.85	229	47.8
MACE-OFF24-M	35.8	53.4	20.6	35.7	10.9	9.43	3.75	217	48.3
ORB-v2	15.3	117	45.4	32.9	12.6	35.4	23.0	115	47.8
ORB-v3	13.7	14.0	21.3	27.1	8.43	16.5	2.06	59.7	**20.4**
TorchMD-Net/ET	81.8	140	84.8	46.8	48.2	54.6	8.60	185	81.2
UAIQM/noBase	39.0	7.19	22.5	24.2	17.3	18.3	22.6	130	35.2
UAIQM/ODM2*	20.6	2.79	9.00	24.5	6.18	4.47	NA	25.2	13.2
UAIQM/GFN2^b^	34.7	8.76	21.7	4.95	7.30	18.8	15.1	22.5	16.7
AIQM1	16.2	11.7	5.44	29.1	13.3	3.69	NA	32.1	15.9
PM6-ML	8.24	14.6	5.77	5.12	3.21	7.27	3.99	15.5	**7.96**
PM6	14.2	35.5	18.6	16.9	12.3	9.94	10.9	44.7	20.4
ODM2*	31.6	13.7	11.5	40.6	8.35	8.99	NA	52.3	23.9
GFN2-xTB	32.4	9.07	11.6	14.6	15.9	10.3	3.21	31.8	16.1
B3LYP	11.7	4.76	6.15	10.2	2.16	1.90	2.67	3.98	**5.44**

a Avg is the average MUE for these 8 reactions. ODM2* is a QM semiempirical method.^12^ PM6-ML, PM6, GFN2-xtb and B3LYP repeat results from Table 2 but only for the subset of 8 reactions.

b UAIQM/GFN2-xTB.

**Table 4:** Root Mean-Squared Deviations (RMSD, in Å) of Geometries Obtained after Constrained Optimization^a^

	Chemical group								
Method	–NH_3_	COOH	^+^CNH_2_	=NH	PhOH	Q	–SH	Zundel3	Avg
MNDO	0.135	0.631	0.181	0.026	0.218	0.235	0.213	0.205	0.231
pddgMNDO	0.031	0.173	0.248	0.036	0.200	0.234	NA	0.092	0.145
AM1	0.018	0.249	0.153	0.033	0.095	0.069	0.044	0.073	0.092
PM3	0.018	0.064	0.057	0.029	0.013	0.060	0.058	0.039	**0.042**
pddgPM3	0.024	0.090	0.062	0.030	0.013	0.078	0.076	0.073	0.056
RM1	0.020	0.069	0.079	0.024	0.015	0.051	0.062	0.033	**0.044**
PM6	0.013	0.068	0.071	0.025	0.021	0.108	0.092	0.066	0.058
PM6-ORG	0.015	0.078	0.080	0.015	0.115	0.128	0.025	0.022	0.060
PM6-D3H4	0.021	0.053	0.079	0.072	0.079	0.106	0.065	0.039	0.064
PM7	0.027	0.034	0.045	0.026	0.032	0.068	0.291	0.069	0.074
noSCC	0.023	0.056	0.101	0.020	0.039	0.154	0.109	0.015	0.065
DFTB2	0.021	0.038	0.045	0.015	0.023	0.149	0.077	0.031	0.050
DFTB3	0.012	0.053	0.058	0.015	0.032	0.169	0.048	0.049	0.055
GFN1-xTB	0.022	0.024	0.042	0.011	0.021	0.137	0.029	0.014	0.038
GFN2-xTB	0.014	0.020	0.027	0.013	0.014	0.110	0.030	0.014	**0.030**
GFN2-xTB^b^	0.090	0.025	0.112	0.055	0.060	0.110	0.208	0.241	0.113
UAIQM^b^ ^,^ ^c^	0.066	0.011	0.064	0.084	0.066	0.095	0.206	0.319	0.114
ANI-1xnr^b^	0.644	0.217	0.372	0.192	0.145	0.352	NA	NA	0.321
PM6-ML^b^	0.096	0.035	0.053	0.056	0.071	0.036	0.237	0.245	0.103
PM6-ML	0.006	0.007	0.021	0.007	0.007	0.018	0.012	0.040	0.015
BLYP	0.011	0.048	0.044	0.044	0.027	0.074	0.026	0.071	0.043
PBE	0.008	0.047	0.036	0.052	0.036	0.081	0.015	0.073	0.044
M06L	0.006	0.030	0.006	0.005	0.011	0.038	0.032	0.046	**0.022**
B3LYP-3G	0.021	0.059	0.178	0.050	0.061	0.145	0.105	0.081	0.088
B3LYP	0.004	0.030	0.015	0.035	0.023	0.027	0.014	0.056	0.026
ωB97X	0.002	0.027	0.027	0.004	0.010	0.024	0.020	0.043	**0.020**

a The initial step of the subset of neutral reactions (as in Table 3) was used, but with the Zundel3 reaction instead of WaterWire. The donor–acceptor distance was constrained (Table 1). Avg is the average RMSD for these 8 reactions.

b Unconstrained optimization.

c UAIQM/GFN2-xTB.

**Table 5:** MUE (kJ/mol) of Relative Energies Calculated with a Hybrid QM/MM Potential for Microsolvated Neutral Reactions Carboxylate1 (COOH), Guanidine2 (^+^CNH_2_), Imidazole2 (=NH) and Phenol1 (PhOH) Described in the QM Region with Additional Water Molecules in the MM Region^a^

	Chemical group				
Method	COOH	^+^CNH_2_	=NH	PhOH	Avg
AM1	28.4	39.9	19.5	27.2	28.8
PM3	39.6	27.0	18.7	33.8	29.8
pddgPM3	22.1	25.2	24.8	22.6	23.7
RM1	3.71	24.7	10.6	4.52	**10.9**
PM6	25.4	12.6	21.6	23.1	20.7
DFTB2	2.82	19.1	12.3	5.22	9.86
DFTB2-NH	2.82	8.51	10.3	5.22	**6.71**
DFTB3	4.13	22.8	11.5	6.67	11.3
DFTB3-NH	4.13	37.2	25.8	6.67	18.4
GFN1-xTB	5.16	9.87	30.8	7.01	13.2
GFN2-xTB	4.18	20.2	7.23	3.41	8.75
UAIQM/GFN2-xTB	4.44	13.5	15.4	5.47	9.71
PM6-ML	17.6	9.31	9.51	3.11	9.88
BLYP	9.80	14.9	24.7	8.12	14.4
PBE	11.1	14.3	24.2	10.5	15.0
M06L	0.73	13.5	11.5	1.32	6.76
B3LYP-3G	20.6	21.2	30.0	22.6	23.6
B3LYP	2.18	11.1	11.7	1.55	6.63
ωB97X	2.05	15.0	3.59	2.65	**5.82**

a Avg is the average MUE for these 4 reactions.

Reference MP2 energies for microsolvated reactions (Table 5 and Figure 2) were obtained with full QM treatment, in which all molecules in the cluster were described as QM centers.

A wide range of approximate and empirically parametrized electronic-structure methods and machine learning (ML) potentials were tested. The first set of methods is based on molecular orbital theory and the neglect of diatomic differential overlap (NDDO) approximation.^7,31^ The following parametrizations were tested: the initial MNDO set;^31^ AM1 ^32^ PM3 ^33^ and RM1 ^34^ with additional Gaussians in the core-repulsion term; pddgMNDO and pddgPM3 with the pairwise directed Gaussian modification;^35^ PM6 ^36^ and PM7 ^25^ also with pairwise corrections and improved parameter sets; PM6-D3H4 ^37^ which adds empirical dispersion (D3) and hydrogen-bond (H4) corrections to PM6; and the recent PM6-ORG^26^ reparametrization for organic and protein molecules. The orthogonalization-corrected ODM2* modified method^12^ was also tested.

The second set of methods tested is based on tight-binding (TB) approximations to Density Functional Theory (DFT). The following methods and parameters combinations were explored: noSCC corresponding to zeroth-order nonself-consistent TB^7,9^ with MIO (mio-1–1) parameters; DFTB2 corresponding to second-order self-consistent-charge (SCC) with MIO parameters^10,38^ and DFTB2-NH with N–H binding corrections (miomod-nh-0-1);^39^ DFTB3 corresponding to third-order SCC with 3OB (3ob-3-1) parameters^40,41^ and DFTB3-NH with N–H corrections (3ob-nhmod-1-2); and the extended tight binding model (xTB) with the GFN1-xTB^27^ and GFN2-xTB^28^ parametrizations.

The third set of approximate methods includes machine-learning potentials. Standalone and general-purpose potentials from the ANI “family” were tested: ANI-1ccx,^11^ ANI-1x-D4 ^42^ and ANI-2x-D4 ^43^ with dispersion corrections,^44^ and ANI-1xnr^13^ parametrized for chemical reactions. More recent standalone ML models MACE-OFF23 (L, large), MACE-OFF24 (M, medium),^45^ and ORB versions 2 ^46^ and 3 (conservative-inf-omat weights)^47^ were also evaluated.

Additionally, ML potentials were applied as corrections (Δ-learning) to baseline electronic-structure methods in AIQM1 (with a ODM2* baseline, release 20211202),^12^ UAIQM^48^ with the coupled-cluster target and based in GFN2-xTB (release 20250115) and ODM2* (release 20240308) QM methods, or without a baseline (noBase), and in the PM6-ML method.^49^ PM6-ML combines the NDDO-based semiempirical QM method PM6 with a short-ranged ML potential based on the TorchMD-Net equivariant transformer (ET) model.^50^

DFT was also tested at multiple levels: generalized-gradient (GGA) with functionals BLYP^51,52^ and PBE;^53^ meta-GGA with M06L;^54^ hybrid functional with B3LYP^52,55^ and range-separated functional with ωB97X-D3.^56,57^ The 6-31G­(d) basis-set^58^ was employed for all functionals, except for calculations denominated B3LYP-3G where the B3LYP functional was used with a smaller 3-21G basis-set. Dispersion corrections (D3-BJ)^44^ were added to all functionals except M06L, which includes dispersion implicitly.

A hybrid QM/MM potential^2^ was used in calculations for the microsolvated clusters (Table 5 and Figure 2). This partition is often used to simulate PT reactions in biological systems.^3,59^ The organic molecule and the reactive central water (Table 1) were described in the QM region, while surrounding water molecules were treated in the MM region. Full electrostatic QM/MM embedding was used. Parameters for the MM region and the Lennard-Jones contribution in the QM region were taken from force-fields CHARMM36m^60^ for organic molecules and TIP3P^61^ for water.

MP2 and DFT calculations were obtained with ORCA version 5,^62^ DFTB calculations were run with DFTB+ release 24.1,^63^ PM6-ORG, PM6-D3H4 and PM7 calculations were conducted with MOPAC version 23,^64^ all other pure NDDO semiempirical and QM/MM calculations, and all geometry optimizations (except for those with methods implemented only in MOPAC, see below) were conducted with pDynamo version 3,^65^ interfaced with ORCA and DFTB+ for the respective QM calculations. For the purpose of this work, the PM6-ML method was implemented in the pDynamo library, interfacing it with TorchMD-Net^50^ for the ML correction and with simple-dftd3 ^66^ for the D3 dispersion contribution. MACE-OFF and ORB computations were conducted with ASE version 3.25.^67^ All other calculations with ML potentials were run with MLatom version 3 via the XACS cloud platform.^68^

It should be noted that software installation was generally straightforward across all packages. Precompiled binaries were available for most QC programs (ORCA, DFTB+ and MOPAC). All other codes are Python-based and could be readily installed from online repositories (GitHub) or through standard Python package managers, along with any required additional libraries. While we used MLatom via a cloud platform for this study, local installation is also supported. The pDynamo and ASE codes configure interfaces to external ML models and QC codes during installation, requiring only subsequent download of the specific ML model weights. Documentation is comprehensive for the QC programs, but more limited for Python-based codes, typically due to their active development. However, all packages provide sufficient examples of basic calculations within their documentation or installation packages to facilitate usage.

### Model Reactions and Geometries

A set of 30 isolated model reactions representing possible proton transfers relevant to biochemical and bioenergetic processes involving protein side chains, quinone substrates, and water molecules was studied here. Table 1 lists the reactions and Figure S3 indicates the proton donor (D) and acceptor (A) atoms, and their molecular orientation. Additionally, proton transfer from water to methyl-phosphate dianion (CH_3_–O–PO_3_^2–^ + H_2_O ⇌ CH_3_–O–PO_3_H^–^ + HO^–^) was evaluated as a test case for doubly anionic species with biological relevance. All reactions had a singlet electronic state, except for Quinone5 that was a doublet.

The distance between proton donor and acceptor atoms (shown in Table 1) was restrained in all reactions at the distance found in the fully optimized geometry for the reactant complex, except for the Eigen and Zundel cations which had the D–A distance frozen at selected values. For reactions Carboxylate3 and 4, where two water molecules are present, two O···O distances were frozen (Figure S3).

Proton transfer pathways are composed of *N* steps or geometries obtained by restraining the D–A distance (Table 1) and the combined difference distance to the transferred proton, ξ = *d*(DH) – *d*(AH). Harmonic potentials with force constant of 1000 kJ mol^–1^·Å^–2^ were used to restrain the D–A distance and to scan ξ with 0.1–0.2 Å increments. Geometries were optimized with the fast inertial relaxation engine (FIRE)^69^ minimizer up to a root mean squared gradient tolerance of 0.1 kJ mol^–1^·Å^–1^ in initial full optimizations and 1 kJ mol^–1^·Å^–1^ in restrained optimizations. These convergence criteria are enough to produce stable and accurate geometries within the approximate quantum-chemical methods used here. PM6 and RM1 methods were used for all optimizations, except when testing reoptimizations for a particular method (Table 4). Reoptimizations with PM6-ORG, PM6-D3H4 and PM7 methods were conducted with MOPAC, with the BFGS algorithm.^70^

Benchmarking along full reaction pathways, rather than only at stationary points, provides a more complete evaluation of method performance. Energies along the reaction coordinate reflect the shape and curvature of potential surfaces, similar to the information available in force-matching approaches.^71^ This is relevant for condensed-phase simulations, where enhanced sampling methods typically explore the entire coordinate range from reactant to product.^15,72^

Relative energies (Table 2) were calculated for the full set of reaction steps (total of 267 geometries, Table 1). Dipoles (Table S1) were calculated for all reaction steps of the following set of neutral reactions: Amine2, Carboxylate1, Guanidine2, Imidazole2, Phenol1, Quinone1, Thiol1 and WaterWire. Geometries (Table 4) were reoptimized only for the initial step of each reaction in the same set of neutral reactions, except for the WaterWire which was exchanged by the Zundel3 reaction.

Microsolvated clusters were prepared for reactions Carboxylate1, Guanidine2, Imidazole2 and Phenol1 each with 4 additional water molecules (except for Phenol1, with 3 additional waters). Geometries were obtained with the RM1 method and the same optimization procedure described above.

The following notation is used for the chemical groups in all tables: amine is −NH_3_, carboxylate is COOH, guanidine is ^+^CNH_2_, imidazole is =NH, phenol is PhOH, quinone is Q and thiol is −SH. Zundel, Eigen and WaterWire water cluster reactions were collectively denoted as H_2_O.

The full set of optimized reaction steps with 267 geometries and MP2 reference energies, microsolvated geometries and additional tables with molecular properties were deposited online^73^ to enable full reproduction of this study.

Deviations in energies and dipole moments are reported as mean unsigned errors (MUEs). When the error is given for a single reaction (Tables 3, 5, and S1), it corresponds to the mean over the *N* steps along the transfer pathway (Table 1). The initial step (reactant state) of each reaction is set as the zero for relative energies. When more than one reaction is presented for the same chemical group (Table 2), the MUE is averaged across all reactions within that group. For Table 4, root mean-squared deviations (RMSD) are computed only for the initial step of each reaction in comparison to the corresponding MP2 geometry. The average MUE (or RMSD for geometries) across all reactions presented in a given table is reported as “Avg” (all Tables and Figure 1).

**Figure 1:**
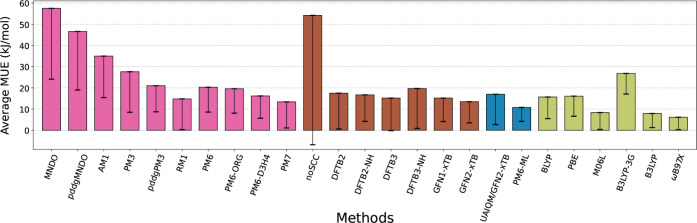
Average mean unsigned error (MUE, in kJ/mol) of calculated relative energies in comparison to the MP2 reference for the complete set of isolated proton transfer reactions (Table 1). The error bar shows standard deviation of MUE across all reactions. Colors distinguish methodological approximations: molecular orbital NDDO methods in pink, DFT tight-binding in brown, ML-corrected (Δ-learning) approaches in blue, and DFT functionals in khaki.

## Results and Discussion

### Energy Benchmark for the Complete Set of Isolated Reactions

The performance of various quantum-chemical methods and hybrid ML-potentials in energy calculations for the complete set of isolated proton transfer reactions (Table 1), including both charged and neutral systems, is shown in Table 2 and in Figure 1.

Semiempirical NDDO methods show a clear evolution in accuracy from early to modern parametrizations. The more recent models like PM7 achieve good accuracy (average MUE = 13.4 kJ/mol), especially for imidazole reactions, where its performance surpasses the DFT workhorse functional B3LYP. RM1 shows the second-best general performance (average MUE = 14.8 kJ/mol). Notably, PM6-ORG, a reparametrization specific for organic and biochemical systems, does not significantly improve upon standard PM6.

As typical for approximate methods, performance varies across chemical groups, with most parametrizations performing poorly for thiols except RM1, which conversely shows relatively weaker performance for phenol and quinone reactions.

Tight-binding methods strongly benefit from the SCC approach. GFN2-xTB (average MUE = 13.5 kJ/mol) and DFTB3 (average MUE = 15.2 kJ/mol) show the best performance, with DFTB3 excelling particularly for carboxylates, thiols and water clusters, in line with previous comparisons of related proton transfer reactions.^21^ However, both DFTB2 and DFTB3 inherit the known deficiency of DFT methods in treating N–H bond energetics,^22,74^ which remains uncorrected in most cases (except for DFTB2 with amine reactions).

Evaluating ML approaches for the full reaction set proved challenging. Standalone potentials like ANI-1ccx and UAIQM/noBase lack an explicit treatment of the net molecular charge in their parametrization, and some ML models failed to complete calculations for charged species when using the MLatom program suite. Consequently, only two Δ-ML methods could be benchmarked in the complete set.

PM6-ML emerges as particularly accurate (average MUE = 10.8 kJ/mol), showing the best performance among all methods more approximate than meta-GGA DFT. It improves upon pure PM6 for all chemical groups and delivers outstanding results for amines and phenols (superior to all methods tested, including DFT).

In contrast, UAIQM/GFN2-xTB shows limited success, only improving upon pure GFN2-xTB for water cluster reactions while degrading performance for all other chemical groups.

The DFT comparison reveals that range-separated functionals (ωB97X) achieve the best accuracy (average MUE = 6.16 kJ/mol), though at significantly higher computational cost. On the other hand, this functional underperforms both M06L and B3LYP for the WaterWire reaction, for which ωB97X has a MUE = 21.5 kJ/mol.

The standard deviation of MUE across all reactions serves as an indicator of method generality and consistency. The most accurate methods—ωB97X, B3LYP, and PM6-ML—exhibit both the lowest average MUEs and smallest dispersions (5.9, 6.7, and 6.6 kJ/mol respectively). Other approximate methods show significantly greater variability: GFN1-xTB, GFN2-xTB, and PM6 (10.0, 11.1, and 11.5 kJ/mol respectively), while PM7, DFTB3, and particularly RM1 demonstrate even higher dispersions (12.3, 12.5, and 14.5 kJ/mol respectively).

The strong performance of PM6-ML when averaged across chemical groups warrants a more detailed analysis. Among the 30 reactions tested, only Quinone4 does not benefit from the ML correction. This reaction involves an aromatic-hydronium cation hydrogen-bond, a class of interaction not represented in the Δ-ML training set.^49^

Reactions involving multiply charged species, such as the biologically relevant proton transfer to anionic phosphate, present a stringent test for approximate potentials. As shown in Figure S1, only NDDO-based methods specifically reparametrized for phosphate chemistry, namely CHOPS^14^ and AM1d-Phot^75^ successfully reproduce the MP2 reference. PM6-ML underperforms here, which can again be attributed to limited hydrogen-bonded phosphate interactions in the Δ-learning training set.^49^

Overall, these findings suggest that PM6-ML is the most broadly applicable among the approximate methods tested here, and that its performance in proton transfer reactions could be further improved by incorporating more hydrogen-bonded species into the Δ-learning training set. Other approximate methods may still be used for modeling specific organic functional groups that exhibit consistently low MUEs (Table 2).

### Performance for Neutral Reactions

A subset of 8 neutral proton transfer reactions was selected to assess ML potentials without the net-charge issues mentioned above (Table 3). Results varied significantly across methods, with standalone ANI potentials performing poorly for most chemical groups, except for carboxylates in the ANI-2x-D4 and ANI-1xnr models.

More recent ML potentials, such as MACE-OFF and ORB-v2, also perform poorly. ORB-v3 shows significant improvement across many chemical groups and achieves the best performance among the standalone ML models. However, its overall accuracy remains well below that of DFT and is comparable to that of approximate semiempirical QC methods. All ML standalone potentials were particularly inaccurate for the WaterWire reaction, likely due to the zwitterionic nature of this system (Table 1).

The Δ-ML approaches based on approximate QC methods showed significantly higher accuracy. PM6-ML continued to achieve the best performance among the more approximate methods (average MUE = 7.96 kJ/mol) and rivals the B3LYP functional. Notably, the same ML model used to correct PM6 (TorchMD-Net/ET, average MUE = 81.2 kJ/mol) performs poorly when trained in the same data set and used as a standalone model^49^ underscoring the critical role of the QC baseline for describing proton transfer reactions.

UAIQM variants exhibited mixed success: UAIQM/ODM2* (average MUE = 13.2 kJ/mol) significantly enhanced its baseline ODM2* (average MUE = 23.9 kJ/mol), particularly for carboxylate and phenol reactions, while UAIQM/GFN2-xTB (average MUE = 16.7 kJ/mol) struggled to improve upon its already accurate GFN2-xTB baseline, showing gains for imidazole but deteriorating performance for guanidine and water cluster reactions. Notably, these latter reactions involve zwitterionic species, again suggesting that ML corrections should be specifically parametrized for them.

AIQM1, while using the same ODM2* baseline as UAIQM/ODM2*, showed intermediate accuracy (15.9 kJ/mol) between the more recent UAIQM corrections, suggesting that incremental improvements were obtained within the UAIQM framework. The standalone UAIQM/noBase (35.2 kJ/mol) performed comparably to ANI-2x-D4 (34.9 kJ/mol), with both showing errors similar to early semiempirical methods like AM1. These results demonstrate that standalone ML potentials remain unsuitable for modeling proton transfer reactions, favoring instead Δ-learning or hybrid ML/QM approaches.

Table 4 shows that the accuracy of geometry optimization varies significantly across methods, with PM6-ML showing the best overall performance (RMSD = 0.015 Å), even surpassing DFT ωB97X (0.020 Å) in constrained optimizations. Except for the early parametrizations (MNDO and AM1), pure semiempirical and tight-binding methods perform similarly, while GFN2-xTB (0.030 Å) shows a better performance.

Many methods show high errors particularly for quinones (Q) and thiols, while PM6-ML maintains consistent accuracy across all functional groups. These trends mirror the patterns for energy accuracy in Table 2, confirming that geometry optimization quality correlates strongly with energetic performance.

We were unable to run constrained optimizations with the MLatom suite, thus only unconstrained results were obtained with methods ANI-1xnr and UAIQM/GFN2-xTB. For a clear comparison, we also rerun PM6-ML and GFN2-xTB optimizations without constraints. Again, PM6-ML performs best, similarly to GFN2-xTB and its UAIQM correction. ANI-1xnr performs worst, confirming this sort of standalone ML potential is not ready to model proton transfer reactions.

Dipole moments were calculated as an example of molecular property relevant to condensed-phase reactivity. Table S1 shows ωB97X (0.34 D) as most accurate, with PM6 (0.39 D) and PM7 (0.38 D) matching DFT performance. GFN2-xTB (0.50 D) comes behind, rivaled by RM1 (0.54 D), in line with previous comparisons of related proton transfer species.^40^

WaterWire and thiol reactions remain challenging, though PM6 and PM7 maintain robust accuracy across all groups. The dipole precision obtained with these two methods suggests they are better models of intermolecular interactions. None of the tested ML approaches or specialized QM corrections (PM6-D3H4, PM6-ML, DFTB2-NH, DFTB3-NH) modify the dipole predictions of baseline methods, thus such methods were not included in Table S1.

Across the previous energy and geometry benchmarks, PM6-D3H4 shows only moderate performance. Inclusion of D3H4 corrections improves noncovalent interactions and hydrogen bonding,^37^ but the method still suffers from the underlying limitations of PM6 in proton transfers. Overall, both PM6-D3H4 and the new PM6-ORG parametrization do not significantly outperform plain PM6, and are clearly less accurate than PM6-ML.

B3LYP-3G gives poor performance across most chemical groups, despite the use of a standard and reliable DFT functional. The minimal basis set (3-21G) limits the description of both polarization and hydrogen bonding, which are critical in proton transfer reactions. This illustrates that basis set choice is a key determinant for DFT approaches and that possible gains in computational speed by using smaller sets should be carefully considered.

### Hybrid QM/MM Calculations in Microsolvated Environments

To evaluate the performance of methods in proton transfer reactions solvated by water, a subset of four neutral reactions was selected (Table 5). Microsolvated models were prepared with additional explicit water molecules to capture solvent interactions with reactive centers. Only methods that showed competitive performance in the isolated reactions were tested. Methods only available in MOPAC (PM6-ORG, PM6-D3H4 and PM7) were not included due to the different^76^ QM/MM implementation in this program, which omits NDDO-like off-diagonal terms in the QM/MM electrostatic interaction, leading to inaccurate computed properties.^2,65^

As expected, Table 5 shows that the DFT functionals M06L, B3LYP and ωB97X yield the highest accuracy, with average MUEs in the range of 6–7 kJ/mol. However, these values are only slightly better than those obtained with the most accurate approximate methods, thus the performance gap is smaller than in the tests for isolated reactions (Table 2). This indicates that inclusion of explicit solvation by QM/MM interactions introduces an additional layer of approximation, which tends to level off errors across different QM methods.

Legacy semiempirical models such as AM1 and PM3 produce high MUEs, while RM1 performs clearly better, particularly for carboxylate and phenol reactions. This trend is consistent with results for isolated reactions. The DFTB2-NH model shows surprisingly good accuracy, especially for the guanidinium reaction, whereas the N–H correction in DFTB3 degrades performance for this reduced test set.

Among the ML-corrected models, PM6-ML yields a substantial improvement over its PM6 baseline for all chemical groups (average MUE reduced from 20.7 to 9.88 kJ/mol), confirming its transferability to solvated systems. In contrast, UAIQM/GFN2-xTB shows no overall improvement compared to the already good performance of the GFN2-xTB baseline, except for the guanidinium case, which remains a particularly challenging reaction across all methods based on DFT.

While accurate dipole moments generally correlate with lower errors in solvation energies,^37,77^ it is difficult to disentangle this effect from the quality of the underlying QM energy description in QM/MM potentials. For example, PM6 shows accurate dipole moments (Table S1) but only modest performance for solvated reactions (Table 5). Conversely, DFTB2-NH performs well in solvated systems despite limited dipole accuracy, likely due to error compensation in its reaction energetics. PM6-ML, which improves significantly over PM6 in gas-phase reaction energies, also shows enhanced performance in microsolvated reactions. Overall, while dipole moments contribute to the quality of solute–solvent interactions, accurate intrinsic energetics appear to be the primary driver of performance in QM/MM simulations.

Figure 2 illustrates the QM/MM potential energy profiles for the microsolvated proton transfer from water to methyl-guanidine (reaction Guanidine2 in Table 1), forming the guanidinium cation and hydroxide. This charge separation reaction displays the largest MUE values in Table 5 and poses a difficult test for all methods.

**Figure 2:**
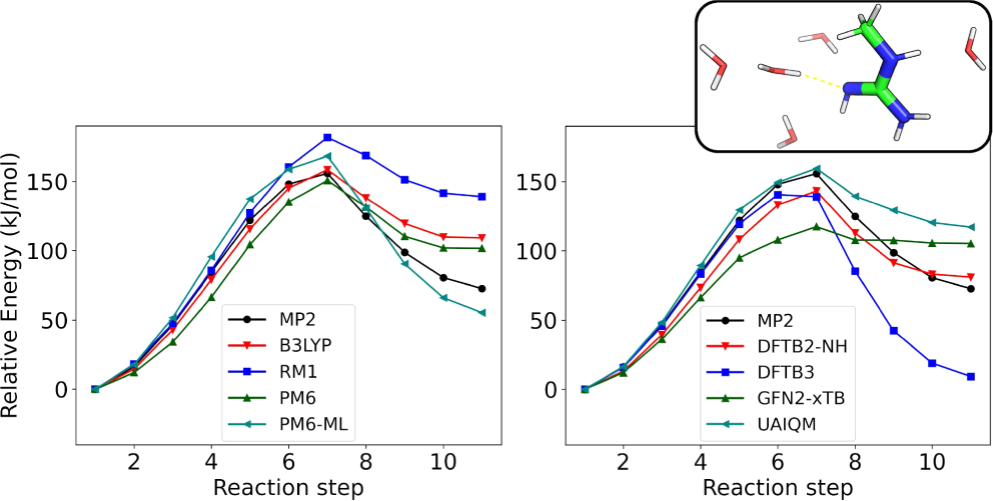
Relative QM/MM potential energy profiles obtained with various QM methods (in the legend, with UAIQM = UAIQM/GFN2-xTB) for the protonation of methyl-guanidine (Guanidine2 in Table 1) microsolvated by water. The inset shows the initial configuration and the proton transfer coordinate, indicated by yellow ticks. The organic molecule and its hydrogen-bonded reactive water were included in the QM region, while the remaining water molecules were treated in the MM region.

B3LYP accurately captures the barrier but overestimates the energy of the guanidinium product by nearly 40 kJ/mol. RM1 shows deviations (>25 kJ/mol) across the entire profile. PM6 reproduces the barrier and the first half of the reaction reasonably but destabilizes the product, an error largely corrected by PM6-ML. However, PM6-ML worsens the barrier, indicating a limitation of the ML correction which was not parametrized for reactive species.

DFTB2-NH provides the most balanced description, yielding the lowest MUE for this reaction (Table 5). In contrast, DFTB3 (or DFTB3-NH) and GFN2-xTB underestimate both the barrier and the product stability. UAIQM/GFN2-xTB improves the first half of the reaction but fails to correct the product state, leaving a significant residual error.

In conclusion, the performance of approximate methods for solvated proton transfer reactions generally reflects their behavior in isolated systems, with the best-performing method varying across chemical groups. Among the NDDO semiempirical methods, RM1 and PM6 offer reasonable accuracy, while GFN2-xTB, DFTB2-NH, and DFTB3 stand out among tight-binding approaches. Machine learning corrections are particularly effective and play a key role in improving baseline methods that show larger errors. Notably, these corrections have proven transferable to proton transfer reactions—despite not being included in their original training sets^48,49^ —and remain effective in QM/MM simulations where only the QM description is corrected by the Δ-learning contribution.

### Additional Considerations: Calculation Speed, Energy Conservation and Stability

While semiempirical QM methods are inherently less accurate than DFT and *ab initio* approaches, their substantially reduced computational cost underlies their widespread use in simulations of chemical reactions, especially when extensive configurational sampling is required.^2,4,59^

To evaluate the computational cost of selected methods, a representative hybrid QM/MM system mimicking the first proton transfer step in the Q_o_ site of cytochrome *bc*_1_ was simulated. This reaction involves proton transfer from the quinol substrate (QH_2_) to a histidine side chain via a bridging water molecule. These groups were included in the QM region (43 atoms), while the remainder of the full system (15,769 atoms total) was treated at the MM level. This reaction has been recently studied,^72^ and all scripts and data used here are openly available.^78^ Methodological details match those used for the QM/MM calculations of the microsolvated reactions. Benchmarks were performed on an AMD © EPYC 7513 processor in serial mode (1 core unless otherwise stated), with 8 GB of allocated memory.

In molecular dynamics (MD) simulations, where both energies and gradients are calculated, the PM6 method completes a 1 ps trajectory in approximately 200 s, corresponding to a throughput of 0.432 ns/day. The PM6-ML variant increases computational cost by only 25%, requiring 250 s/ps (0.345 ns/day). At this rate, a 1 ns trajectory, often sufficient for convergence of condensed-phase free energy profiles,^17,59,72^ can be sampled in about 3 days of wall-clock time.

In contrast, DFT-based QM/MM simulations are around 3 orders of magnitude slower. For the same system, serial QM/MM simulations with M06L and B3LYP require 107,300 s/ps (0.0008 ns/day) and 309,500 s/ps (0.0002 ns/day), respectively. Parallelization across 16 CPU cores (the practical scaling limit for this system) reduces these to 12,580 s/ps (0.0069 ns/day) for M06L and 26,260 s/ps (0.0033 ns/day) for B3LYP, still 2 orders of magnitude slower than PM6. This highlights the significant sampling advantage offered by approximate QM methods in hybrid QM/MM simulations.

Increasing the QC region size from 43 to 116 atoms yields 1 ps MD trajectories in 1310 s (0.066 ns/day) for PM6 and 1424 s (0.061 ns/day) for PM6-ML. Thus, PM6 exhibited the characteristic steep scaling of QC methods (albeit with a small prefactor), while the ML correction maintains linear scaling, consistent with previous reports.^49^

Energy conservation was also assessed using MD simulations in the NVE ensemble (Figure S2), using the leapfrog integrator available in the pDynamo3 library.^65^ Both PM6 and PM6-ML maintained a stable total energy over relatively long time scales, confirming the suitability of these methods for extended MD simulations.

Convergence of the self-consistent field (SCF) procedure is another practical concern. Empirically, RM1 was prone to SCF failures and occasionally produced unphysical geometries, for example, incorrect protonation of carbon atoms in phenol rings. In contrast, PM6 was found to be more robust, with no convergence issues observed even in large systems. DFTB methods, particularly when applied to large QM regions, also showed convergence instabilities, as previously reported.^49^

Finally, the training requirements of approximate methods should be considered. Semiempirical QC methods benefit from their underlying physical symmetries and interaction laws, which result in models with much smaller number of parameters and allow for effective parametrization using significantly smaller training data sets than those required for ML approaches. Therefore, the computational and human costs associated with generating and curating large data sets must be accounted for in the development (both initial training and fine-tuning) of ML potentials. Notably, the training of deep ML frameworks has become feasible largely due to automatic differentiation,^79^ which enables more stable and systematic parameter optimization, as well as finer control when tuning models for specific reactions or chemical processes. These advantages of automatic differentiation are now also becoming available for the optimization of approximate QC methods.^80^

## Conclusions

This study provides a comprehensive benchmark of semiempirical and tight-binding quantum chemical methods, and machine-learning potentials for modeling proton transfer reactions relevant to biochemical systems. The results demonstrate that the more recent semiempirical models such as RM1, PM6 and PM7, as well as tight-binding models like GFN2-xTB, DFTB3 and DFTB2-NH, can achieve reasonable accuracy, particularly when selected for specific functional groups. However, none of them is applicable generally, without prior validation.

Machine-learning corrections applied through Δ-learning improve the accuracy of baseline methods, especially PM6-ML, which consistently ranks among the most accurate approaches across both isolated and microsolvated reactions. Although no reactions were included in its training set, the correction proved transferable to proton transfer processes, apparently whenever information on the relevant hydrogen bonds was present in the training data. PM6-ML also remained effective in hybrid QM/MM potentials where the ML correction is applied only to the QM region.

In contrast, standalone ML potentials performed poorly and failed to generalize to proton transfer reactions, indicating that such models are not yet suitable for modeling this class of chemical transformations.

DFT methods were the most accurate among the methods tested here, but are computationally costly. Approximate methods like PM6-ML enable simulations with sampling times at least 2 orders of magnitude longer than those accessible with DFT, while maintaining good energetic and structural accuracy. These findings support the use of modern ML-enhanced (Δ-learning) semiempirical methods as robust, efficient alternatives for simulating proton transfer reactions in complex biochemical environments.

Intrinsic (gas-phase) reaction energies remain the primary determinant of performance for approximate quantum methods. However, it is important to note that the errors (MUEs) reported here for isolated reactions do not directly translate to errors in free energy profiles for condensed-phase QM/MM simulations. In solvated environments, reaction barriers and energies for charge-separation are often significantly reduced. As such, deviations observed in the gas-phase are expected to be mitigated and should be considered upper bounds for the errors in condensed-phase simulations. Furthermore, the QM/MM framework introduces an additional layer of approximation that tends to level off differences in accuracy across QM methods.

Promising future directions include applying Δ-ML strategies to correct short-range QM/MM potentials, which could improve the description of interactions between QM and MM regions. Additionally, automatically differentiable semiempirical and tight-binding methods^80^ may enable both improved parametrization and rapid fine-tuning for proton transfer reactions.

## Supplementary Material



## Data Availability

Reference (MP2) geometries and energies, and spreadsheets with isolated energies and dipoles were deposited online^73^ to enable full reproduction of this study. All software used here is freely available online: pDynamo,^81^ ORCA,^62^ DFTB+,^63^ MOPAC,^64^ MLatom,^68^ ASE,^67^ and TorchMD-Net.^50^

## Notes

One table with molecular dipoles and three figures showing profiles for PT to phosphate, energy conservation in QC/MM simulations, and the orientation of reactants and donor/acceptor centers for PT reactions (PDF)
