# Supplementary material for: Benchmark of Approximate Quantum Chemical and Machine Learning Potentials for Biochemical Proton Transfer Reactions
Source: J Chem Theory Comput. 2025 Jun 30;21(14):7149–59. doi: 10.1021/acs.jctc.5c00690 (PMC12288007; doi:10.1021/acs.jctc.5c00690)

# Supporting Information

## Benchmark of approximate quantum chemical and machine learning potentials for biochemical proton transfer reactions

Guilherme M. Arantes* and Jan Řezáč

*Instituto de Estudos Avançados, Universidade de São Paulo, Rua da Praça do Relógio 109, 05508-050, São Paulo, SP, Brazil; Instituto de Química, Universidade de São Paulo, Av. Prof. Lineu Prestes 748, 05508-900, São Paulo, SP, Brazil; Institute of Organic Chemistry and Biochemistry, Czech Academy of Sciences, 160 00 Prague, Czech Republic*

E-mail: garantes@iq.usp.br

Contents:

- Table S1: MUE of molecular dipoles for neutral reactions.
- Figure S1: Profiles for proton transfer from water to methyl-phosphate dianion.
- Figure S2: Conservation of total energy in QC/MM simulations.
- Figure S3: Orientation of reactants and donor/acceptor centers for all reactions.

Table S1: Average MUE (in debye) of molecular dipoles for the subset of neutral 8 reactions (as in Table 3). Avg is the average dipole MUE for these 8 reactions and bold font is used to indicate the best performance within each group of methods.

| | Chemical group | | | | | | | | Avg |
|---|---|---|---|---|---|---|---|---|---|
| Method | $-NH_3$ | COOH | $^+CNH_2$ | =NH | PhOH | Q | −SH | $H_2O$ | |
| MNDO | 0.78 | 0.47 | 1.24 | 0.70 | 0.61 | 1.01 | 0.48 | 1.40 | 0.84 |
| pddgMNDO | 0.55 | 0.38 | 1.04 | 0.72 | 0.50 | 0.97 | NA | 1.11 | 0.75 |
| AM1 | 0.54 | 0.17 | 0.98 | 0.51 | 0.48 | 0.75 | 0.47 | 1.04 | 0.62 |
| PM3 | 0.93 | 0.20 | 1.08 | 0.46 | 0.63 | 0.71 | 0.54 | 1.34 | 0.74 |
| pddgPM3 | 0.73 | 0.20 | 0.97 | 0.39 | 0.61 | 0.51 | 1.06 | 1.32 | 0.72 |
| RM1 | 0.51 | 0.10 | 0.80 | 0.32 | 0.53 | 0.72 | 0.49 | 0.88 | 0.54 |
| PM6 | 0.57 | 0.13 | 0.31 | 0.35 | 0.23 | 0.44 | 0.50 | 0.57 | **0.39** |
| PM6-ORG | 0.26 | 0.24 | 0.73 | 0.24 | 0.55 | 0.26 | 0.76 | 0.69 | 0.47 |
| PM7 | 0.66 | 0.22 | 0.27 | 0.29 | 0.25 | 0.40 | 0.45 | 0.51 | **0.38** |
| noSCC | 1.82 | 0.49 | 2.79 | 2.87 | 0.78 | 0.84 | 1.11 | 1.46 | 1.52 |
| DFTB2 | 1.11 | 0.60 | 0.81 | 1.20 | 0.57 | 0.95 | 0.64 | 1.21 | 0.89 |
| DFTB3 | 0.75 | 0.19 | 0.26 | 1.10 | 0.46 | 0.99 | 0.51 | 0.65 | 0.61 |
| GFN1-xTB | 0.69 | 0.33 | 0.68 | 0.64 | 0.70 | 1.24 | 0.48 | 0.28 | 0.63 |
| GFN2-xTB | 0.32 | 0.26 | 0.49 | 0.34 | 0.48 | 1.33 | 0.53 | 0.28 | **0.50** |
| BLYP | 2.95 | 0.16 | 1.35 | 0.83 | 0.70 | 0.30 | 1.28 | 0.08 | 0.96 |
| PBE | 2.74 | 0.15 | 1.29 | 0.81 | 0.62 | 0.30 | 1.32 | 0.12 | 0.92 |
| M06L | 1.68 | 0.18 | 0.94 | 0.63 | 0.41 | 0.33 | 1.15 | 0.19 | 0.69 |
| B3LYP-3G | 2.49 | 0.18 | 1.37 | 0.81 | 0.59 | 0.53 | 1.13 | 0.76 | 0.98 |
| B3LYP | 1.65 | 0.13 | 0.99 | 0.63 | 0.41 | 0.20 | 0.69 | 0.11 | 0.60 |
| $\omega$B97X | 0.49 | 0.11 | 0.68 | 0.49 | 0.25 | 0.21 | 0.24 | 0.23 | **0.34** |

Figure S1: Relative potential energy profiles for proton transfer from water to methyl-phosphate dianion: $CH_3$-O-$PO_3^{2-}$ + $H_2O \rightleftharpoons CH_3$-O-$PO_3H^-$ + $HO^-$. Calculations were performed using multiple computational methods as described in the main text, including the semiempirical QC methods specifically reparametrized for phosphate reactions, CHOPS[14] and AM1d-Phot.[75]

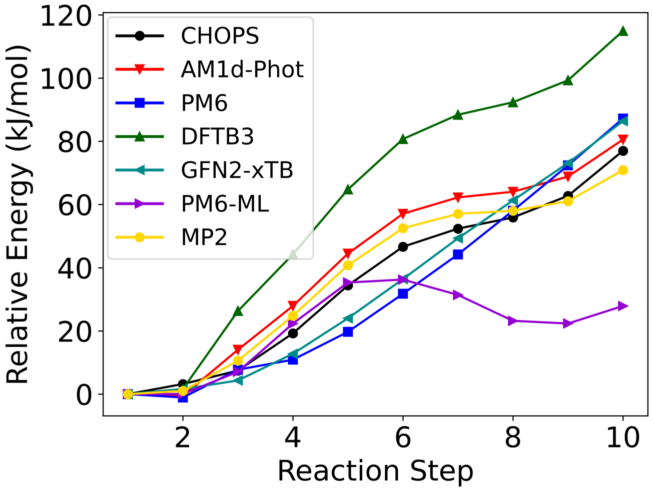


Figure S2: Conservation of total energy (potential + kinetic) during a molecular dynamics simulation in the NVE ensemble, with a hybrid QM/MM potential using a PM6 or a PM6-ML description of the QM region. The enzymatic proton transfer catalyzed by cytochrome $bc_1$ was used as an example,[72] as described in the main text. The observed energy fluctuations (present also in pure MM simulations) arise primarily from the droplet molecular model used with frozen atoms in the external shell, and can be reduced by decreasing the MD integration time-step.

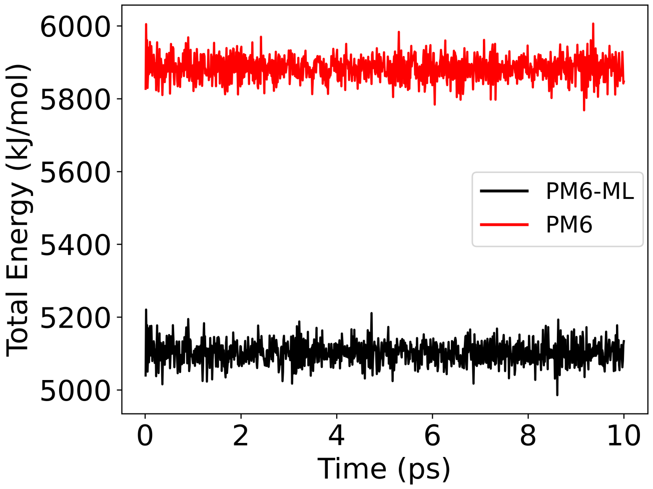

Figure S3: Orientation of reactants for the complete set of proton transfer reactions studied here. Contacts shown as black dashes connect the reactive (donated) proton and acceptor atoms. Red dashes in species with two or more water molecules denote an H-bond free to relax (not scanned or restrained) during optimizations.

**Amine:**

1) 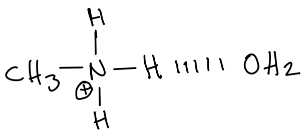

2) 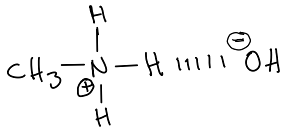

**Carboxylate:**

1) 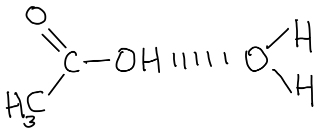

2) 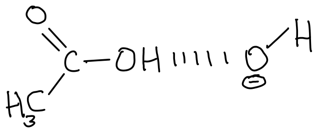

3) 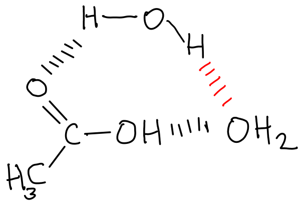

4) 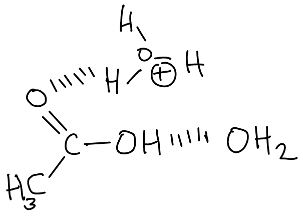

**Guanidine:**

1) 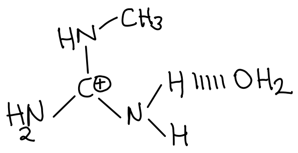

2) 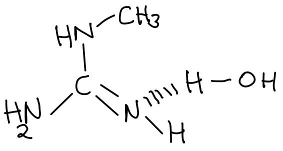

3) 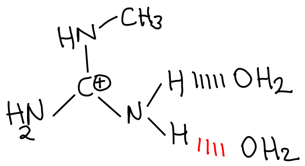

**Imidazole:**

1) 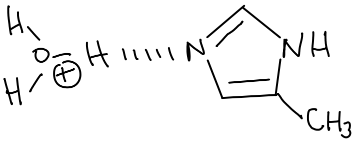

2) 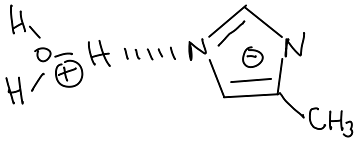

3) 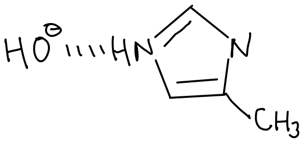

4) 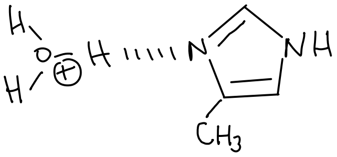

**Phenol:**

1) 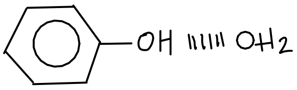

2) 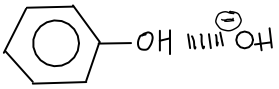

3) 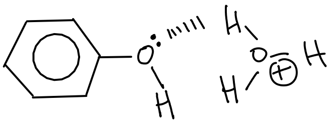

**Quinone:**

1) 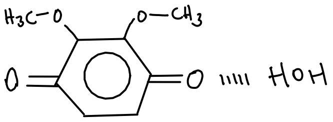

2) 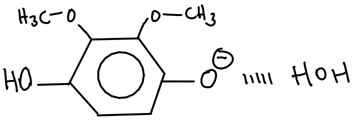

3) 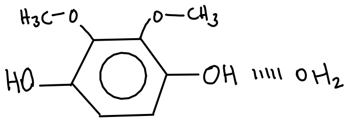

4) 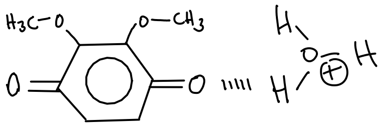

5) 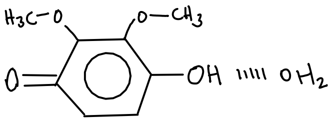

**Thiol:**

**1)** 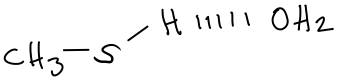

**2)** 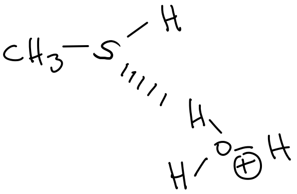

**Water wire:**

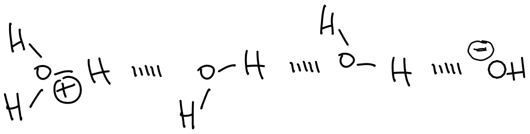

**Eigen ion:**

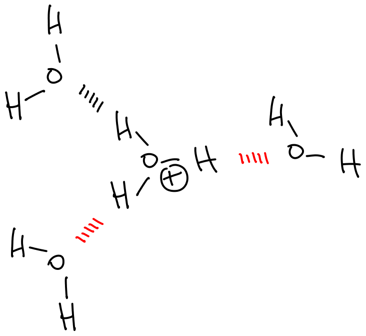

**Zundel ion:**

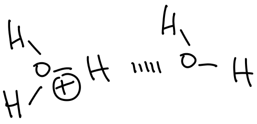

Supplement: Supplementary file 1 [file ct5c00690_si_001.pdf]
